# Growth, Biomass Partitioning, and Photosynthetic Performance of Chrysanthemum Cuttings in Response to Different Light Spectra

**DOI:** 10.3390/plants11233337

**Published:** 2022-12-01

**Authors:** Moein Moosavi-Nezhad, Boshra Alibeigi, Ahmad Estaji, Nazim S. Gruda, Sasan Aliniaeifard

**Affiliations:** 1 Photosynthesis Laboratory, Department of Horticulture, Aburaihan Campus, University of Tehran, Pakdasht P.O. Box 33916-53755, Iran; 2 Department of Horticultural Science, North Carolina State University, Raleigh, NC 27695, USA; 3 Department of Horticultural Sciences, Campus of Agriculture and Natural Resources, University of Tehran, Karaj P.O. Box 31587-77871, Iran; 4 Department of Horticultural Sciences, Faculty of Agriculture, Vali-E-Asr University of Rafsanjan, Rafsanjan P.O. Box 77188-97111, Iran; 5 Department of Horticultural Science, INRES–Institute of Crop Science and Resource Conservation, University of Bonn, 53121 Bonn, Germany

**Keywords:** controlled-environment agriculture, chlorophyll fluorescence imaging, OJIP transient, light quality, propagation

## Abstract

Chrysanthemum (*Chrysanthemum morifolium*) is among the most popular ornamental plants, propagated mainly through stem cuttings. There is a lack of information regarding the impact of the lighting environment on the successful production of cuttings and underlying mechanisms. The light spectrum affects plant morphology, growth, and photosynthesis. In the present study, chrysanthemum, cv. ‘Katinka’ cuttings, were exposed to five lighting spectra, including monochromatic red (R), blue (B) lights, and multichromatic lights, including a combination of R and B (R:B), a combination of R, B, and far red (R:B:FR) and white (W), for 30 days. B light enhanced areal growth, as indicated by a higher shoot mass ratio, while R light directed the biomass towards the underground parts of the cuttings. Monochromatic R and B lights promoted the emergence of new leaves. In contrast, individual leaf area was largest under multichromatic lights. Exposing the cuttings to R light led to the accumulation of carbohydrates in the leaves. Cuttings exposed to multichromatic lights showed higher chlorophyll content than monochromatic R- and B-exposed cuttings. Conversely, carotenoid and anthocyanin contents were the highest in monochromatic R- and B-exposed plants. B-exposed cuttings showed higher photosynthetic performance, exhibited by the highest performance index on the basis of light absorption, and maximal quantum yield of PSII efficiency. Although R light increased biomass toward roots, B light improved above-ground growth, photosynthetic functionality, and the visual performance of Chrysanthemum cuttings.

## 1. Introduction

Chrysanthemum (*Chrysanthemum morifolium*), also called mums or chrysanths, belongs to the Asteraceae family and is among the most popular ornamental plants worldwide. With more than 40 recognized species, mums dominate the floriculture market, ranking second after the rosa [1,2]. Chrysanthemums are generally propagated through stem cuttings, especially in developing countries lacking tissue culture equipment. Cuttings are an easy method of propagation, yet they are prone to adverse environmental conditions in the early stages. Therefore, they need to be propagated in controlled environments.

Controlled-environment agriculture (CEA) is growing worldwide. Due to intensive production methods and more extended cropping periods, the yield in CEA is much higher than in the open field. In addition, CEA products usually have high quality and high market value, even in sub-optimal climates and geographies, which justifies the extra costs for investment and operation [3,4]. Therefore, besides greenhouse production, producers are inclined to benefit from a more attractive method of crop production called indoor vertical farming under light-emitting diodes (LED). These methods with artificial lights have been referred to as VFS (i.e., vertical farming systems [5]), PFAL (i.e., plant factory with artificial lights [6]), LVS (i.e., LED-equipped vertical systems [7]), or CPPS (i.e., closed plant production systems [8]). 

Cutting propagation is generally practiced under greenhouse conditions, leading to several problems. The main difficulties comprise the inability to fully control environmental conditions such as temperature, relative humidity, and light, as well as the low production per unit of area (caused by the horizontal pattern of cultivation) [7], the greater possibility of pest attacks, and the lower successful cutting ratio.

Most greenhouses are covered with polyethylene, glass, polycarbonate or nylon. Heat exchange through these covers is noticeable, thereby decreasing the insulation rate, making the inside conditions more reliant on the outside environment. Accordingly, the temperature inside greenhouses fluctuates over different seasons and even day and night [9,10,11]. Maintaining an optimum intensity of light in greenhouses is another challenge. The inside light intensity at the crop level depends on the intensity of natural light, which fluctuates over days and seasons. An elevation in light intensity leads to an increase in greenhouse temperature. This can cause excessive leaf transpiration, which adversely affects cuttings in the first days of propagation as the adventitious roots have not yet developed.

Indoor farms are highly insulated, mainly built by brick walls, leading to reliable and fixed environmental conditions. Moreover, the growing pattern is vertical, thereby increasing the production per unit of area and decreasing overall energy consumption per unit of production [7].

Moosavi-Nezhad et al. (2022) comprehensively assessed grafted seedling production in a greenhouse and a vertical farm from an energy, environmental, and economic point of view. The authors reported that grafted seedling production in vertical farms leads to an environmentally friendly production approach with a decrease in energy consumption. Also, they reported a 24% increase in net profit for seedling production on a vertical farm with only five floors.

Indoor propagation of cuttings seems reliable, but the only obstacle might be the lack of natural light, making producers turn to the use of artificial lights. Despite all the benefits of plant production in vertical farms, comprehensive research into the required artificial light is needed. Light is one of the most imperative environmental cues, regulating plant behavior and affecting its development [6]. Since the source of energy for photosynthesis in the indoor environment is artificial light, manipulating the indoor environment′s lighting properties has an emerging interest for the propagation of plants in the CEA. 

The importance of environmental cues on the successful propagation of horticultural plants when the connection between shoot and root is limited has been strongly emphasized [9,12]. Among them, light properties play a crucial role. The primary light properties which affect plant integrity include duration (photoperiod), intensity, and spectrum. However, high light intensities are not suggested for cutting propagation since cuttings are newly-cut stems from the mother plant and have no extensive root system to support the subsequent transpiration. Thus, moderate light intensities (≈50–300 photosynthetic photon flux density (PPFD), which varies based on plant species and crop density, may be used. 

The quality (spectrum) of the lighting environment in CEA greatly influences crops′ growth, morphology, and physiological responses. The wavebands that chlorophyll pigments mostly absorb are in the range of R and B lights. Therefore, R and B light spectra have been widely used in CEA to study growth, morphology, and different aspects of physiology in different plant species [13,14,15,16]. Light spectrum in the range of 600–700 nm (R) is primarily involved in the growth and development of plants. However, when R light is the sole source of light for plants, it causes physiological and morphological disorders such as photosynthetic disturbances, leaf curling, and epinasty [9,14,17]. On the other hand, light spectrum in the range of 400–500 nm (B), has been reported as the waveband promoting the development of chloroplast, formation of chlorophyll, production of pigments, and promotion of photosynthesis; however, when B light is the sole source of light, the growth of plants would be primarily restricted [18]. Therefore, to prevent the negative impacts of monochrome lights, di-chrome or multi-chrome lights are recommended for indoor CEA systems. 

Light quality significantly affects plants′ morphology, as an essential aspect of their marketability in the agricultural industry. For instance, monochromatic R led to the downward curling of the leaves [9,19], negatively affecting marketability. In contrast, B induced more pigmentation, which is positive for marketing. Besides absorbing the energy of the light photons, mainly by the chlorophyll pigments, light also induces morphological alterations in plants, called photomorphogenesis, which is devised due to the morphological response of plants to the light. Phytochromes are the primary photoreceptors that absorb the light energy of the R and Far-red (FR), and they modulate the expression of different genes related to photomorphogenesis. Cryptochromes and phototropins are B light photoreceptors. Cryptochromes are mainly involved in determining plant height, flowering time, and circadian rhythms [20]. Phototropins participate in the movement and rearrangement of chloroplasts, light harvesting in the photosynthesis system, and reducing photodamage [21].

Overall, it seems that the impact of light quality on the propagation of plants needs further investigation. The underlying mechanisms involved in the response of propagules to light quality are still in their infancy. In the present study, we employed five light spectra to produce Chrysanthemum cuttings in a controlled environment to evaluate light quality effects on cutting survival ratio, growth and morphology, photosynthetic performance and pigmentation.

## 2. Results

Following 30 days of exposure to different light spectra, including red (R), red and blue (R:B), red, blue and far-red (R:B:FR), white (W) and blue (B: see the spectrum in Figure 9), morphological parameters, pigments, photosynthetic performance, and soluble carbohydrates of Chrysanthemum cuttings were assessed. 

### 2.1. Survival, Growth, and Morphology of Chrysanthemum Cuttings

The survival ratio of cuttings was 100%, irrespective of light-quality treatments. Cuttings under monochromatic R and B lights produced more leaves (≈13 and ≈12, respectively) than those exposed to multichromatic lights (Table 1). However, the average individual leaf area (i.e., leaf area leaf^−1^; Table 1) was largest in plants under multichromatic lights (i.e., R:B, R:B:FR, and W). R-, R:B- and R:B:FR- exposed cuttings produced the most extended shoots and roots compared to those produced under W and B lighting conditions.

**Figure 1:**
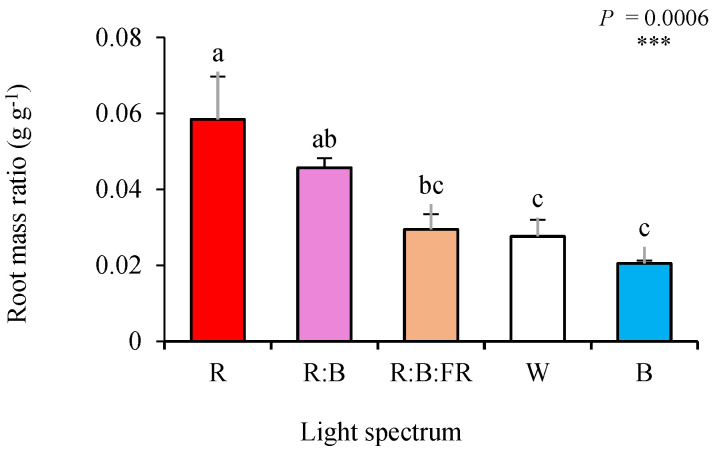
Root mass ratio of chrysanthemum cuttings exposed for 30 d to different light quality regimes (red (R), red and blue (R:B), red, blue and far-red (R:B:FR), white (W) and blue (B); see the spectrum in Figure 9). During the experiment, the photosynthetic photon flux density was set to 150 ± 5 µmol m^−m^ s^−s^. Six replicates per treatment were assessed. Columns with the same letters are not significantly different at *p* ≤ 0.05, according to Duncan’s multiple range tests. Bars represent SEM.

The highest areal biomass (shoot FW and DW; Table 1) was noted in B-exposed plants, followed by those produced under R:B and R:B:FR lighting conditions. The lowest areal biomass was recorded in R-exposed cuttings. On the other hand, B- exposed cuttings showed the lowest underground biomass (root FW and DW: Table 1). This was also obvious when comparing the biomass partitioning to roots and shoots of different lighting treatments. Root mass ratio (root DW/plant DW: Figure 1) increased by exposure to R, while shoot mass ratio (shoot DW/plant DW: Supplementary Figure S1) was elevated by exposure to B light. The relative values of morphological parameters (relative to W as the control) were plotted in a spider plot to facilitate the comparison of light quality regimes (See Supplementary Figure S2A).

### 2.2. Leaf-Soluble Carbohydrate Content

**Figure 2:**
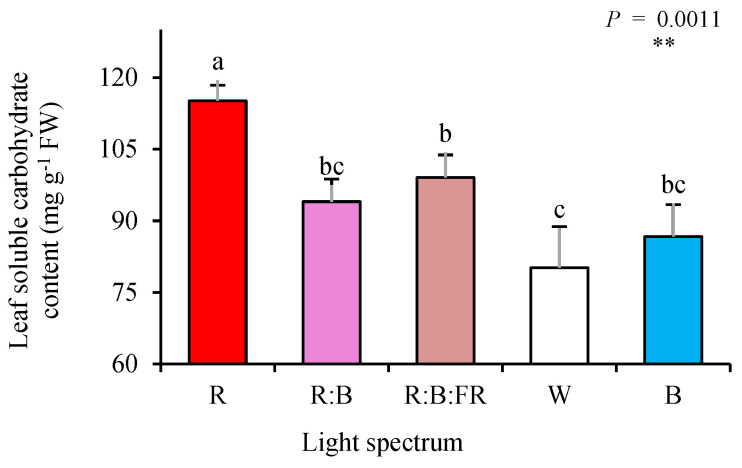
Leaf soluble carbohydrate content of chrysanthemum cuttings exposed for 30 days to different light quality regimes (red (R), red and blue (R:B), red, blue and far-red (R:B:FR), white (W) and blue (B); see the spectrum in Figure 9). During the experiment, the photosynthetic photon flux density was set to 150 ± 5 µmol m^−2^ s^−1^. Six replicates per treatment were assessed. Columns with the same letters are not significantly different at *p* ≤ 0.05, according to Duncan’s multiple range tests. Bars represent SEM.

The highest concentration of soluble carbohydrates was detected in leaves of cuttings exposed to R light, which was 32% higher than the concentration of soluble carbohydrates recorded for plants exposed to other lighting treatments. Among multichromatic lights, exposure to R:B:FR and W led to the highest and lowest carbohydrate concentration, respectively (Figure 2).

### 2.3. Photosynthetic Pigments

**Figure 3:**
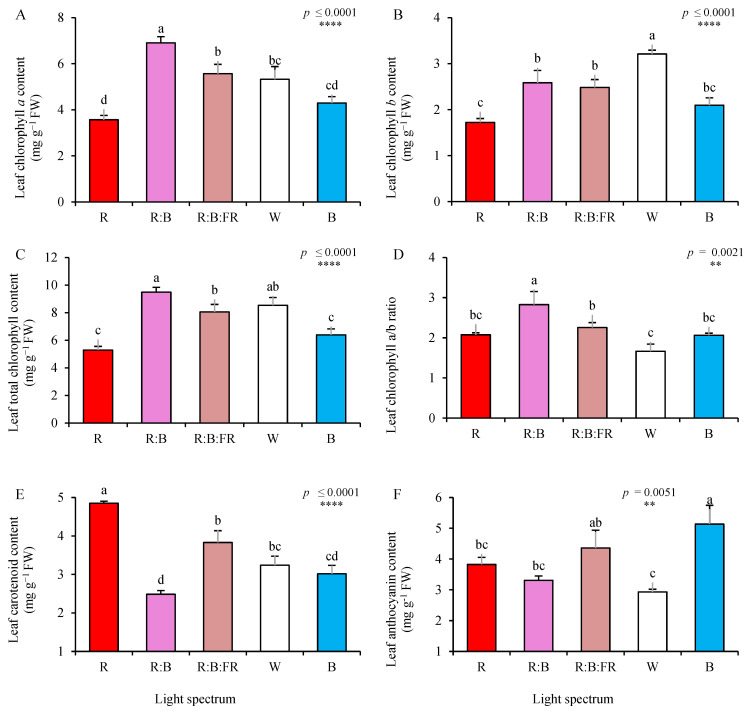
Leaf chlorophyll *a* and *b* (**A**,**B**), total chlorophyll (**C**), chlorophyll *a*/*b* ratio (**D**), total carotenoids (**E**), and total anthocyanin content (**F**) of Chrysanthemum cuttings exposed for 30 days to different light quality regimes (red (R), red and blue (R:B), red, blue and far-red (R:B:FR), white (W) and blue (B); see the spectrum in Figure 9). During the experiment, the photosynthetic photon flux density was set to 150 ± 5 µmol m^−2^ s^−1^. Six replicates per treatment were assessed. Columns with the same letters are not significantly different at *p* ≤ 0.05, according to Duncan′s multiple range tests. Bars represent SEM.

One indication of good quality propagation is leaf greenness, which is one of the essential items of product marketability [9]. Therefore, leaf chlorophyll (Chl), carotenoid, and anthocyanin contents were evaluated at the end of the experiment (Figure 3). Multichromatic light exposures led to a higher production of Chl *a*, Chl *b*, and total Chl compared to the monochromatic lights (Figure 3A–C). Among them, while the highest Chl *a* was recorded in plants exposed to R:B leaves, the highest Chl *b* was recorded in plants exposed to W light. The highest ratio of Chl *a* to *b* (Figure 3D) was noted in plants exposed to R:B.

On the other hand, chrysanthemum cuttings exposed to R showed the highest carotenoids, which was twice the carotenoids content recorded in the leaves of R:B exposed cuttings (4.8 vs. 2.4 mg g^−1^ FW). While R exposure increased carotenoids′ concentration, B light increased the anthocyanins content (Figure 3F). Anthocyanins content of cuttings under B was 25% higher than their content in R-exposed cuttings.

The relative values of different analyzed pigments (relative to W as the control) were plotted in a spider plot to facilitate the comparison of light quality regimes (see Supplementary Figure S2A).

### 2.4. Chlorophyll Fluorescence Imaging

**Figure 4:**
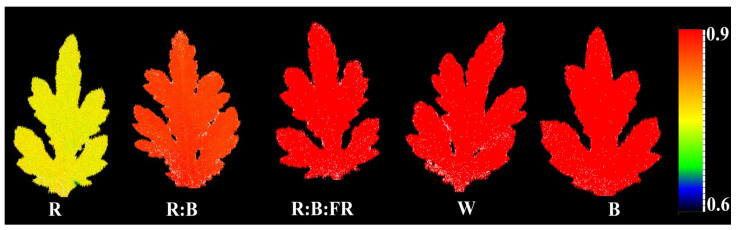
Pseudo-color images of maximum quantum yield of photosystem II (F_v_/F_m_; (equations and explanations in Table 2)) exhibited by leaves sampled from chrysanthemum cuttings exposed for 30 days to different light quality regimes (red (R), red and blue (R:B), red, blue and far-red (R:B:FR), white (W) and blue (B); see the spectrum in Figure 9). During the experiment, the photosynthetic photon flux density was set to 150 ± 5 µmol m^−2^ s^−1^. Six replicates per treatment were assessed.

The effect of lighting conditions during the rooting of chrysanthemum cuttings on overall photosynthetic functionality was assessed by evaluating the spatial pattern of fluorescence emission through pseudo-color images of F_0_, F_m_, and F_v_/F_m_ (Figure 4; equations and explanations in Table 2). R-exposed cuttings showed the highest F_0_ and F_m_ as indicated by the warmer coloring (more redness than blueness; see color guide scale at the right side of the figure). However, the same plants showed the lowest F_v_/F_m_. Both F_0_ and F_m_ decreased in plants under other lighting treatments, with the highest amplification under B. The greatest F_v_/F_m_ was seen in the leaves of B- exposed chrysanthemum cuttings.

### 2.5. Polyphasic Chlorophyll Fluorescence Transient (OJIP) 

**Figure 5:**
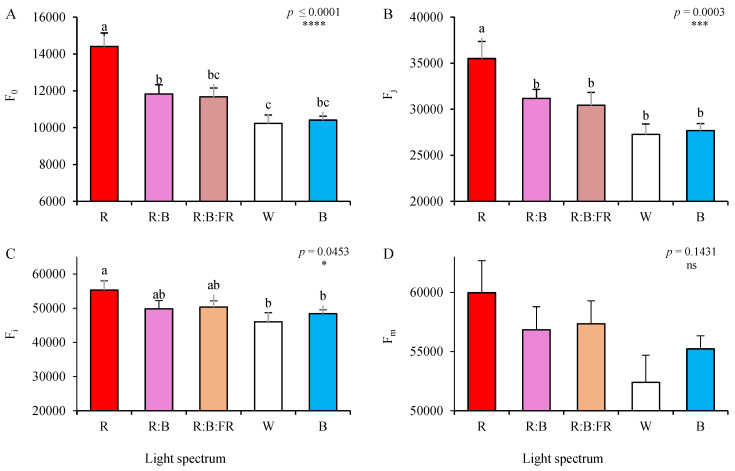
Minimum fluorescence (F_0_; **A**), fluorescence intensity after 2 ms (F_j_; **B**), fluorescence intensity after 30 ms of OJIP (F_i_; **C**), and maximum fluorescence (F_m_, **D**) (equations and explanations in Table 2) of OJIP protocol applied to the leaves of chrysanthemum cuttings exposed for 30 days to different light quality regimes (red (R), red and blue (R:B), red, blue and far-red (R:B:FR), white (W) and blue (B); see the spectrum in Figure 9). During the experiment, the photosynthetic photon flux density was set to 150 ± 5 µmol m^−2^ s^−1^. Six replicates per treatment were assessed. Columns with the same letters are not significantly different at *p* ≤ 0.05, according to Duncan′s multiple range tests. Bars represent SEM.

Transient chlorophyll fluorescence analysis was recorded in the leaves of chrysanthemum cuttings expanded under different lighting conditions. Minimum fluorescence (F_0_; Figure 5A), was the highest in the photosynthetic apparatus of R- exposed cuttings, followed by R:B and R:B:FR plants (see also Figure 4). The lowest F_0_ was recorded in W-exposed cuttings, though with no significant (*p* ≤ 0.05) difference with B- exposed ones (Figure 5A). While the highest value of F_j_ (explanation in Table 2) was noted in plants under R, no statistically significant changes were noted among other lighting conditions (Figure 5B). The highest value of F_i_ (explanations in Table 2) was also recorded in R- exposed cuttings (Figure 5C). In contrast, the changes in the values of F_m_ were not statistically significant at *p* ≤ 0.05 (*p* = 0.1431).

**Figure 6:**
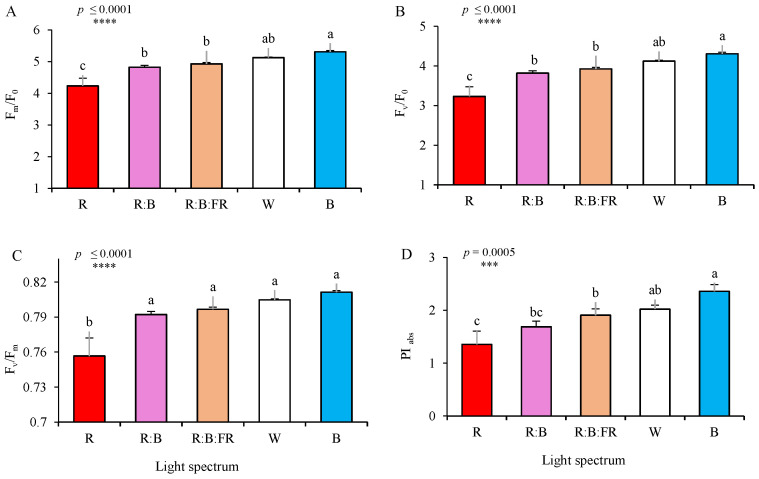
Relative maximal and minimum chlorophyll fluorescence (F_m_/F_0_) (**A**), maximum efficiency of the water oxidation reaction (F_v_/F_0_; **B**), maximal quantum yield of PSII efficiency (F_v_/F_m_; **C**), and performance index on the basis of light absorption (PI _abs_; **D**) (equations and explanations in Table 2) of chrysanthemum cuttings exposed for 30 d to different light quality regimes (red (R), red and blue (R:B), red, blue and far-red (R:B:FR), white (W) and blue (B); see the spectrum in Figure 9). During the experiment, the photosynthetic photon flux density was set to 150 ± 5 µmol m^−2^ s^−1^. Six replicate plants per treatment were assessed. Columns with the same letters are not significantly different at *p* ≤ 0.05, according to Duncan′s multiple range tests. Bars represent SEM.

Following 30 days of exposure to light treatments, the differences in the values of F_m_/F_0_, F_v_/F_0_, F_v_/F_m_, and PI_abs_ were recorded (Figure 6). The lowest and highest values of all the mentioned parameters were recorded in chrysanthemum cuttings exposed to monochromatic R and B lights, respectively. Generally, a gradual rising trend can be seen in all mentioned parameters with increasing the B to R ratio (from 100% R to 100% B). Relative maximal and minimum chlorophyll fluorescence (F_m_/F_0_) and maximum efficiency of the water oxidation reaction (F_v_/F_0_) were the highest under monochromatic B and the lowest under monochromatic R (Figure 6A,B). The maximum quantum yield of photosystem II (F_v_/F_m_; Figure 6C) was only decreased in plants exposed to R, but showed no significant (*p* ≤ 0.05) differences among other lighting treatments. The performance index on the basis of light absorption (PI_abs_; Figure 6D) was the lowest under R with no significant difference with R:B and the highest under B, with no significant difference with W.

**Figure 7:**
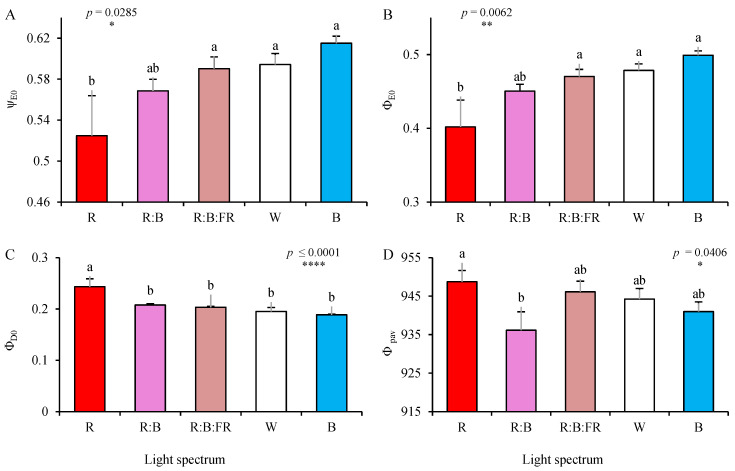
The probability that a trapped exciton moves an electron into the electron transport chain beyond Q^−A^ (ψ_E0_; **A**), Φ_E0_ (**B**), Φ_D0_ (**C**), and Φ_pav_ (**D**; equations and explanations in Table 2) of chrysanthemum cuttings exposed for 30 days to different light quality regimes (red (R), red and blue (R:B), red, blue and far-red (R:B:FR), white (W) and blue (B); see the spectrum in Figure 9). Six replicates per treatment were assessed. Columns with the same letters are not significantly different at *p* ≤ 0.05, according to Duncan′s multiple range tests. Bars represent SEM.

The values of ψ_E0_ and Φ_E0_ (see equations and explanations in Table 2) increased in cuttings exposed to B, with no significant differences with those under W, R:B:FR, and R:B. However, r-exposed cuttings also showed the lowest ψ_E0_ and Φ_E0,_ which was ≈ 20% lower than B-exposed cuttings (Figure 7A,B).

In contrast, the values of Φ_D0_ and Φ_pav_ increased in R-exposed cuttings. The Φ_D0_ in R-exposed cuttings was significantly different from Φ_D0_ of other cuttings exposed to other lighting treatments. The Φ_pav_ of R-exposed cuttings showed only a significant difference with those grown under R:B (Figure 7C,D).

**Figure 8:**
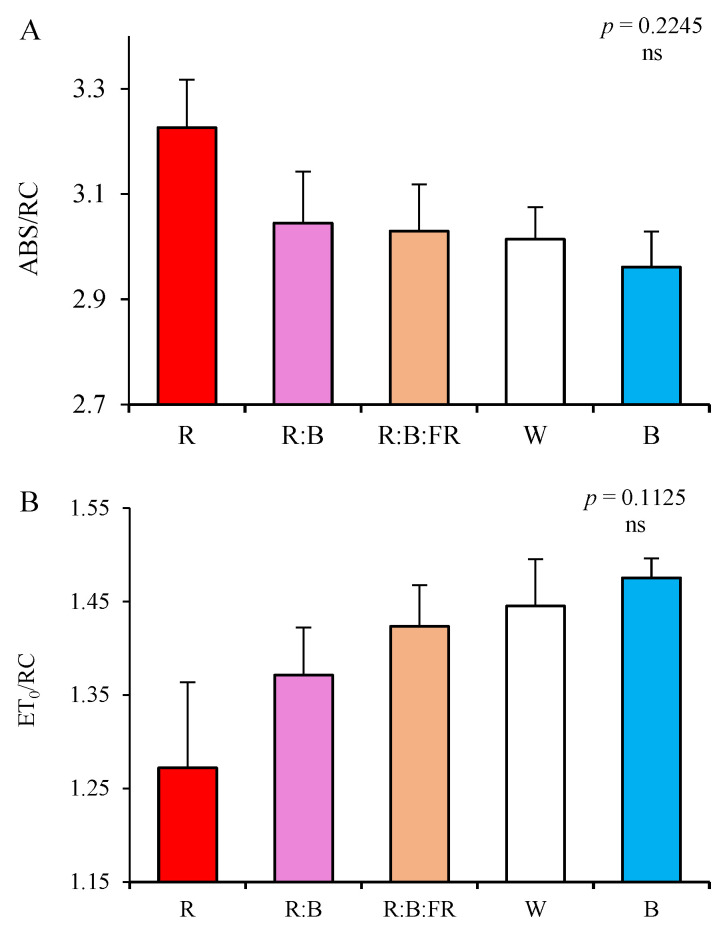

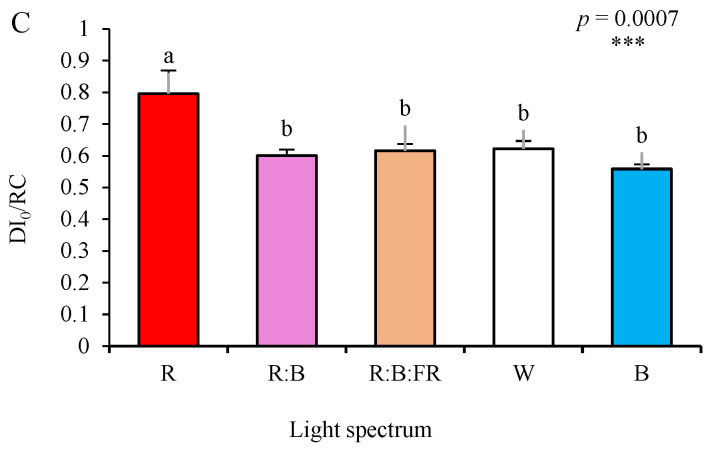
Specific energy fluxes per reaction center (RC) for energy absorption (ABS/RC; **A**), electron transport flux (ET_0_/RC; **B**), and dissipated energy flux (DI_0_/RC; **C**) (equations and explanations in Table 2) of chrysanthemum cuttings exposed for 30 days to different light quality regimes (red (R), red and blue (R:B), red, blue and far-red (R:B:FR), white (W) and blue (B); see the spectrum in Figure 9). Six replicates per treatment were assessed. Columns with the same letters are not significantly different at *p* ≤ 0.05, according to Duncan′s multiple range tests. Bars represent SEM.

Specific energy fluxes (per reaction center; RC) were also influenced following 30 days of exposure to different lighting conditions (Figure 8). Absorption flux (of antenna Chls) per RC (ABS/RC; Figure 8A) increased in the photosynthetic apparatus of cuttings under R, although the differences were not statistically significant (*p* = 0.2245; Figure 8A). The same situation was observed in electron transport flux per RC (ET_0_/RC; Figure 8B). While the highest value was recorded under B, the changes were not significantly different (*p* = 0.1125).

However, dissipated energy flux per RC (DI_0_/RC; Figure 8C) showed a high overall significance level (*p* = 0.0007) among lighting conditions. The value of DI_0_/RC under R was 0.79, which showed a 24 and 30% increase compared to R:B- and B-exposed cuttings.

The relative (relative to W as the control) values of OJIP test parameters were plotted in a spider plot to facilitate the comparison of light quality regimes (see Supplementary Figure S2B).

## 3. Discussion

Light is an indispensable environmental factor influencing plant growth, morphology, and photosynthesis [4,22,23]. Higher plants have evolved to sense not only the intensity and quality of light but also the duration and angle of the incident light. They further use that information to optimize their growth and development concerning the prevailing environmental conditions. Therefore, the ability of plants to maximize their photosynthetic productivity relies on their capacity to perceive, evaluate, and respond to light quality, quantity and direction. When we consider light quality, plants sense wavelengths from ≈380 nm to ≈750 nm via different photoreceptors. Cryptochromes and phototropins confer the detection of ultraviolet A (UV-A) and blue spectra. However, the spectra on the other end of the sensible light spectra, i.e., red and far-red, are detected by the photoreversible phytochrome family of photoreceptors [24,25].

Studying the effects of light qualities on the response of plants can be investigated based on energy or signaling inputs of the light. In the energy aspects, chlorophyll pigments are dominant in the plants′ response, which is mainly reflected in the photosynthetic electron transport of the plants. In the signaling aspects, photoreceptors take the dominant role. The underlying responses are mainly reflected in morphological and anatomical alterations at the plant and cellular levels, triggering the underlying signaling cascades [26,27]. Here, while we do not delve deep into the information on various photoreceptors, as it is contrary to the purpose of this research, we try to discuss the possible reasoning behind the energy-based photosynthetic functionality of the chrysanthemum cuttings.

### 3.1. B Light Enhanced Areal Growth, while R Light Directed the Biomass towards the Underground Parts of Chrysanthemum Cuttings

Light quality can potentially change the allocation of biomass toward the aboveground or underground parts of the plant [28,29]. In the present study, B and R lights caused a contrasting pattern of biomass partitioning into the above- or under-ground parts of the chrysanthemum rooted cuttings. B light directed the highest biomass toward the shoot and led to the highest shoot dry mass. In contrast, R light partitioned more biomass into the root and caused the highest root dry weight to be gained when the biomass of both monochromatic light recipes is compared to the biomass of plants exposed to other light qualities (Figure 1 and Table 1). The alteration of biomass partitioning to different plant organs as a consequence of plant growth in different lighting environments has been reported in diverse plant species such as chrysanthemum [30], saffron [28,31], bromeliads [32], and lettuce [23,33].

Contrary to the findings obtained in the present study, it has been reported that R light is an inducer of shoot growth. In contrast, B light usually restricts biomass allocation to the above-ground parts (especially into the leaves, while it promotes biomass partitioning into the generative organs) [32]. However, Hernández and Kubota (2016) reported that monochromatic B light caused a dramatic increase in the length of cucumber seedlings compared to other spectra, which follows our results [34]. Increasing the above-ground length in the early stages of plant life by monochromatic B light was also reported by Moosavi-Nezhad et al. (2021) in grafted watermelon seedlings [9]. It seems that the impact of B light on the production of tall plants also depends on the plants’ developmental phase [9].

The inductive role of R light and the negative impact of B light on root growth has also been reported before [13]. The possible reasons for contradictory findings for the effects of R and B light on above- and under-ground growth can be related to the different organology and lack (or limited) root-to-shoot connection of the cuttings. It seems that in plant-material samples, when there are limitations between the root and shoot connections (e.g., in the grafted seedlings or in the cuttings), the priority is bridging the connections between the root and shoot organs. In the grafted watermelon seedlings, the B light facilitated the healing and development of the leaves in the scion part, while the R light caused leaf epinasty [9]. In this regard, B light promotes stomatal opening and induces transpirational forces, leading to a stream of water toward the above-ground part of the cutting stems [35]. Above-ground parts of the plants (especially the leaves) take up water and soluble nutrients through transpirational forces. In the case that the evaporative demands by the above-ground parts are higher than the provision of water (negative water balance between above- and under-ground parts) [36], this would result in the wilting of the above-ground parts and the failure of the propagation practice [12]. Since B light did not negatively impact the cuttings′ survival (Table 1), it did not impose a negative water balance between the above-ground and underground parts. Later, due to the facilitation of the transpirational water stream caused the growth of the above-ground parts of the chrysanthemum cuttings. 

### 3.2. Pigmentation and Carbohydrate Levels in the Leaves of Chrysanthemum Cutting Were Influenced by Growing Light Quality

In the present study, R light caused an accumulation of carbohydrates in the leaves of the chrysanthemum cuttings. In contrast, the lowest carbohydrate content was detected in cuttings exposed to B light and those with a high percentage of B in their overall spectrum (W light composed of 35% B (400–500 nm), 49% intermediate (500–600 nm), and 16% R (600–700 nm) and R:B light composed of 50% R and 50% B). This finding follows previous reports showing the promotion of carbohydrate accumulation following exposure to R light. For instance, in grafted watermelon seedlings, R-light induced the accumulation of carbohydrates in the leaves [9]. Furthermore, a negative impact of B-light on carbohydrate accumulation in the leaves and its inductive roles in the underground parts has been reported for saffron plants.

In contrast, the opposite responses for carbohydrate accumulation in the above-ground and under-ground parts have been reported for the saffron exposed to R-light [28]. This occurs due to an imbalance in the loading and unloading of carbohydrates in the source organs (leaves). First, carbohydrates remain in the leaves, and second, their unloading to the sink organs is limited. This phenomenon usually occurs as the result of exposure to monochromatic R light, leading to the accumulation of carbohydrates in the leaves [37,38]. In contrast, the B light facilitates the unloading of carbohydrates from the leaf to the sink organs [28,31]. 

Based on the results obtained in the present study, the highest Chl *a*, *b* and total Chl content were detected in plants exposed to multichromatic lighting treatments. At the same time, monochromatic lights (especially the R light) caused the lowest concentrations of Chls in the leaves of the chrysanthemum cuttings (Figure 3). Hosseini et al. (2019) also showed that basil plants exposed to multichromatic light (especially R:B light) contained more chlorophyll than monochromatic R or B lights. In addition, multichromatic lighting recipes increased the chl *a*/*b* ratio in the present study, following the findings of Dou et al. [38]. The negative impact of R light on the biosynthesis of chlorophyll has been reported for R light because R-exposed plants contain less tetrapyrrole precursor 5-aminolevulinic acid for the biosynthesis of chlorophylls [39]. It has also been reported that an increase in the ratio of chl *a*/*b*, improved the activities of ribulose-1,5-bisphosphate carboxylase (Rubisco) and phosphoenolpyruvate carboxylase and promoted stomatal opening, which improved photosynthesis per unit of leaf area [40].

Carbohydrates participate in a wide range of plant processes, including anthocyanin production. Anthocyanin biosynthesis is a light-dependent process. Environmental cues such as light intensity, the short wavelengths of the visible spectrum, and low temperature are usually used during growth to elevate anthocyanin levels. It has been reported that anthocyanins accumulate following B light exposure. This could be due to the upregulation of anthocyanin biosynthetic genes by the B-light, which causes a decrease in the carbohydrate level as the precursor for the biosynthesis of anthocyanin [41]. On the other hand, R light upregulates phytoene synthase leading to the biosynthesis of β-carotene, while the B light reduces the biosynthesis of β-carotene [42]. In the present study, the highest carotenoid content was detected in R-exposed plants. The highest anthocyanin content was measured in B-exposed plants (Figure 3). A study on *Dunaliella salina* showed that growing plants under R light caused carotenoid accumulation and elevated ROS scavenging capacity, while B light induced a drastic decrease in carotenoid content [43].

### 3.3. B Light Exposure Enhanced while R Light Down-Regulated the Photosynthetic Capacity of Chrysanthemum Cuttings

The spectral absorption range of Chl pigments, as the primary photosynthetic pigment in plants, is in the range of visible light (≈400–700 nm). Chls absorb mainly the energy of the light spectra in the range of B and R lights. However, due to the inhibitory impact of sole R light exposure on photosynthesis and the occurrence of the “red syndrome” [9,18] in the present study, together with R light, two other light qualities, including B and FR, were also used. Following our expectation, in the present study, the negative impact of R light was detected on the electron transport system, which downregulated the biophysical parameters related to the PSII efficiency of the newly emerged leaves on the chrysanthemum cuttings. This can be seen in the negative impact of R light on maximal and minimum chlorophyll fluorescence (F_m_/F_0_), the maximum efficiency of the water oxidation reaction (F_v_/F_0_), the maximal quantum yield of PSII photochemistry (F_v_/F_m_), performance index in light absorption basis (PI_abs_), ψ_E0_ and Φ_E0_ of R light exposed cuttings (Figure 6 and Figure 7). R-disturbed electron transport is the consequence of either damage or down-regulation of the photosystem II (PSII) reaction center (RC). The damage to the PSII RC can be seen through an increase in ABS/RC (Figure 8) and the excitation pressure on the electron transport chain, which resulted in more energy dissipation and lower electron transport in the RC (Figure 8). The negative impact of R light on electron transport system functionality has been extensively studied and reported before [14]. On the other hand, B light, or the spectral treatments containing B-waveband, improved photosynthetic functionality or removed the negative impact of sole R light on the photosynthetic functionality of the leaves of chrysanthemum cuttings. The improving effects of B light or removal of the negative impact of R light when the spectrum containing both light qualities have been reported in different plant species such as cucumber [14,44], basil [13], chrysanthemum [18], carnation [45], watermelon seedling [9], saffron [28,31] and many others.

Although the FR light is believed to be out of the absorption spectra of the Chls, the involvement of FR light in the action spectrum is a matter of debate. Due to low photosynthetic efficiency, FR photons are considered insufficient for driving photosynthesis [46]. However, early studies showed that the photosynthetic rate increased by the co-exposure of photosynthetic samples to photons of R and FR wavelengths [47,48]. In fact, leading scientists are now even arguing about adding FR spectra (≈701–750 nm) to the definition of photosynthetically active radiations (PAR) [49,50]. Zhen and Bugbee (2020) mentioned that FR photons could be equally efficient compared to traditional photosynthetic photons [50]. Furthermore, Zhen and Van Iersel (2017) reported that FR light enhanced the photosynthetic efficiency of shorter wavebands that over-excited PSII [49]. Therefore, in the present study, to improve the photosynthetic functionality as well as to see its impact on the growth and morphology of chrysanthemum cuttings, FR light was used in combination with R and B lights. Furthermore, including FR with shorter wavelength photons provides a balance in excitation energy distribution between PSII and PSI to improve photosynthesis efficiency [49,51]. Accordingly, FR and B light combined with R light improved the photosynthetic functionality in the leaves of chrysanthemum cuttings (Figure 6, Supplementary Figure S2).

In conclusion, based on the findings obtained from the present study, the successful propagation of chrysanthemum through stem cutting strongly depends on the spectrum of the lighting environment. B light exposure force plants to partition more biomass into the above-ground parts (shoot), while R light directs more biomass toward the underground parts (roots). Exposing the cuttings to R light increased carbohydrates in the leaves. For green pigmentations, multichromatic lighting treatments worked better than monochromatic lights. However, R and B light elevated carotenoid and anthocyanin pigmentations in the leaves of chrysanthemum cuttings. Furthermore, the sole application of R light down-regulated the photosynthesis and induced a red-light syndrome, while B light improved photosynthetic performance. Therefore, for the propagation of chrysanthemum cutting, the spectrum of the lighting environment should be manipulated based on the aim of the cutting production.

## 4. Materials and Methods

### 4.1. Plant Material and Growth Media

Stock greenhouse-grown chrysanthemums (*Chrysanthemum morifolium* cv. ‘Katinka’) plants were maintained in the vegetative stage for providing further cutting materials in an experiment conducted at Photosynthesis Laboratory, University of Tehran, Iran (35° 48′12″ N. 51°68′61″ E). Cuttings (unrooted; ≈10 cm long; six leaves; same architecture) were obtained from the same node positions of the stems. Stem cuttings with similar fresh weight (FW), length, and the number of leaves were used. Cuttings were distributed randomly in 15 groups to be further planted in fifteen 24-cell seedling trays filled with a commercial growing mixture containing peat, perlite and coco-peat in a ratio of 7:2:1 (*v/v/v*) [9]. Before cutting, the mixture was first subjected to excessive irrigation with distilled water to reduce the substrate′s electrical conductivity (EC). Newly-planted stem cuttings were first irrigated with distilled water and then transferred to specialized lighting chambers for the subsequent 30 days. Growing media moisture was maintained near maximum water-holding capacity by regular watering. Cuttings were irrigated with half-strength Hoagland and Arnon nutrient solution. Once a week, seedling trays were subjected to excessive watering with distilled water to prevent nutrient accumulation and reduce the growing media’s EC. 

### 4.2. Lighting Treatments

Cuttings were initially exposed to darkness for two days to prevent leaf dehydration and then to five lighting spectra provided by light-emitting diode (LED) modules (Parcham Co, Tehran, Iran). Three 24-cells seedling trays were placed in each lighting chamber, each illuminated with different light spectra, including red (R), red and blue (R:B), red, blue, and far-red (R:B:FR), white (W) and blue (B). Light spectra were monitored using a Sekonic light meter (Sekonic C-7000, Tokyo, Japan) and intensity was adjusted to 150 ± 5 µmol m^−2^ s^−1^ photosynthetic photon flux density (PPFD) at the top of the plant canopy using a PAR-FluorPen FP 100-MAX (Photon Systems Instruments, Drásov, Czech Republic). The applied intensity was higher than in the study of Schroeter-Zakrzewska and Pradita (2021), who used 50 µmol m^−2^ s^−1^ [52] or that used by Zheng and Van Labeke (2017), who employed 100 µmol m^−2^ s^−1^. The application of higher light intensity in the present study was because we used 24-cell seedling trays instead of pots (as it is mainly used for commercial propagation of chrysanthemum cutting), leading to a denser canopy which needs a more intense light. Therefore, using lower light intensities may impose light limitations on the cuttings. In addition, opaque black-white curtains were placed around each light regime treatment to prevent light contamination. 

**Figure 9:**
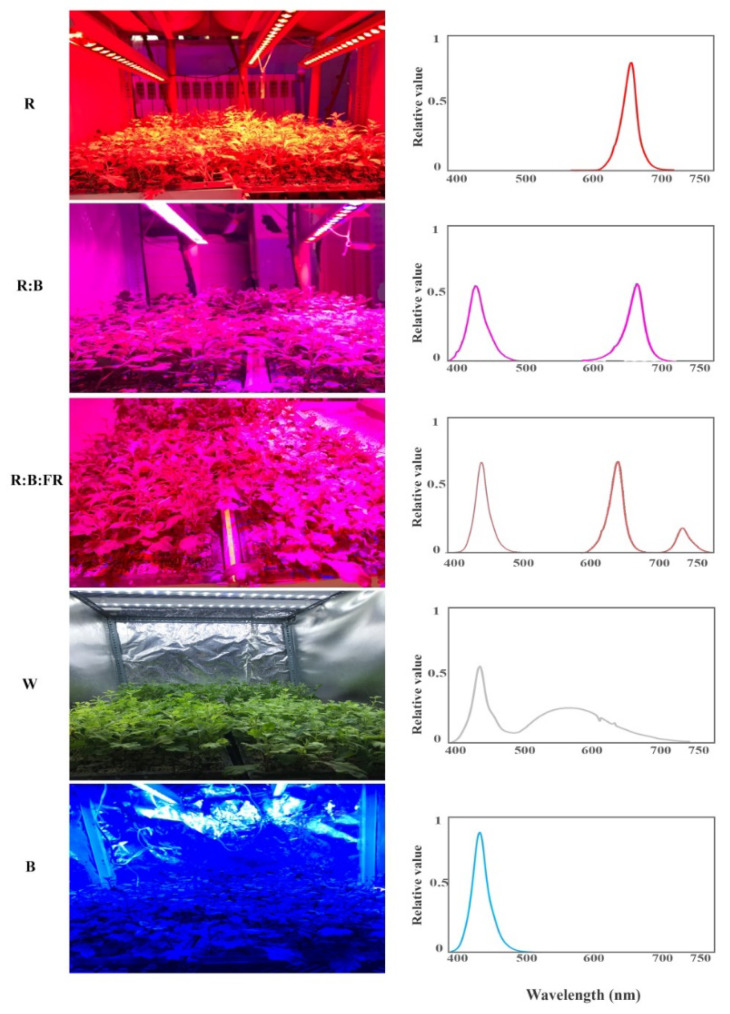
Chrysanthemum cuttings under different light spectra (from top to bottom: red (R), red and blue (R:B), red, blue, and far-red (R:B:FR), white (W) and blue (B)) and relative spectral distribution of each light quality regime (right panel). Measurement of the spectrum was carried out three times (*n* = 3). During the experiment, the photosynthetic photon flux density was set to 150 ± 5 µmol m^−2^ s^−1^.

The day/night temperature and photoperiod were controlled to 26/18 ± 1 °C (temperature controller model: TRB-125D, Shiva Amvaj, Tehran, Iran) and 16/8 h, respectively. The relative humidity of the growth room was maintained at 70% ± 5% throughout the cultivation period. Two ventilation fans (12 V, 0.90A) were installed in each unit to ensure uniform air circulation. All plants were exposed to the same controlled conditions (temperature, irrigation, photoperiod and light intensity, etc.) except for the different light spectra (Figure 9).

Six plants were sampled per light quality regime (two from the middle of each tray). Sampled cuttings were surrounded by border cuttings that were not sampled to minimize border effects.

### 4.3. Morphological and Growth Assessments

The effect of the light regime on cutting growth, morphology, and biomass partitioning was assessed. Evaluations included shoot length, root length, number of leaves, plant and individual leaf area, and under and above-ground dry masses. For leaf area determination, leaves were scanned (HP Scanjet G4010, Irvine, CA, USA), and then their area was calculated using the Digimizer software (version 5.3.5, MedCalc Software, Ostend, Belgium). Plant leaf area was divided by leaf numbers to measure the average individual leaf area. Root length was also measured after removing the substrate from the roots via gentle washing. For measuring dry weight, samples were placed in a forced-air drying oven for 72 h at 80 °C. Subsequently, by using dry mass, root mass ratio (RMR; root mass/plant mass) and shoot mass ratio (SMR; shoot mass/plant mass) were calculated.

### 4.4. Leaf Total Soluble Carbohydrate Content

The colorimetric quantification of leaf total soluble carbohydrates content was employed during the rooting of chrysanthemum cuttings [53]. Anthrone reagent was first prepared in a dark room by dissolving 0.1 g of anthrone (0.2%) in 100 mL of concentrated sulfuric acid (98%). Subsequently, samples (0.1 g) and anthrone reagent (1 mL) were loaded in tubes. The tubes were placed for 15 min in a water bath (90 °C), cooled for 5 min (0 °C) and vortexed for one min. A 20-min heating phase to room temperature (25 °C) was then performed before the final reading. The spectrophotometric absorbance (Optizen pop, Mecasys Co. Ltd. Daejeon, Korea) was recorded at 620 nm. A standard curve based on a series of known glucose concentrations was prepared.

### 4.5. Leaf Pigmentation

#### 4.5.1. Chlorophyll and Carotenoids

The effect of the light regime on photosynthetic pigments (chlorophyll, carotenoids) content was assessed in cutting leaves. Leaf samples were processed flash-frozen in liquid nitrogen immediately after collection. Following fine chopping, portions weighing 0.5 g were homogenized with the addition of 10 mL of 80% acetone. This primary acetone extract was then filtered, and the filtered extract was diluted by adding 2 mL of 80% acetone per mL of extract. Since chlorophyll is light-sensitive, the extraction took place in a dark room [9]. The obtained extract was subjected to reading on a spectrophotometer (Mapada UV-1800; Shanghai. Mapada Instruments Co., Ltd., Shanghai, China). Total chlorophyll and carotenoid contents were calculated [54]. Six leaves were assessed per treatment. Replicate leaves were collected from different individual plants.

#### 4.5.2. Anthocyanins

The light regime effect on leaf anthocyanin was determined. Frozen samples (0.5 g) were extracted in 10 mL of 1% HCL in methanol for 48 h. The liquid extract was separated by centrifugation at 7000× *g* for 5 min. Subsequently, the absorbance of the supernatant was measured at 515 nm.

### 4.6. Chlorophyll Fluorescence Imaging

As a sensitive indicator of the photosynthetic performance of the chrysanthemum cuttings, dark-adapted values of the maximum quantum yield of PSII (F_v_/F_m_; equation in Table 2) were recorded in leaves detached from cuttings exposed to each light spectrum. Measurements were conducted on leaf surfaces using a handy FluorCam (FC 1000-H; Photon Systems Instruments, Drásov, Czech Republic). The leaves were dark-adapted by turning LED lamps off (≥20 min) prior to evaluation. F_v_/F_m_ was then evaluated by applying a saturated PPFD of 3900 µmol m^−2^ s^−1^ [9,55]. Nine leaves were assessed per treatment. Replicate leaves were collected from individual plants.

### 4.7. Polyphasic Chlorophyll Fluorescence Transient (OJIP) Evaluation

The polyphasic chlorophyll fluorescence induction curve (OJIP transient) was obtained in leaves attached to chrysanthemum cuttings exposed to each light spectrum. By employing the OJIP test, the shape changes of the OJIP transient are quantitatively translated to a set of parameters (equations in Table 2), which relate to the in vivo adaptive behavior of the photosynthetic apparatus (particularly PSII) to the growth environment [31,56]. Measurements were conducted on attached leaves using a handy PAR-FluorPen FP 100-MAX (Photon Systems Instruments, Drásov, Czech Republic) following dark adaptation (≥ 20 min). The light intensity employed (3900 μmol m^−2^ s^−1^ PPFD) was sufficient to generate maximal fluorescence for all light-quality treatments.

Following dark adaptation, leaves exhibited a polyphasic chlorophyll fluorescence rise during the first second of illumination. The fluorescence transient plotted on a logarithmic time scale typically includes the following phases: O to J, J to I, and I to P. F_0_ represents the so-called “Open” (O) state of the OJIP transient [57,58] measured at 50 µs. F_0_ primarily originates from the light-harvesting antenna pigments [31,59,60]. F_j_ and F_I_ originate from the inflections at 2 and 30 ms, respectively [61]. On the other hand, F_m_ comes from the reduction-oxidation state of the primary quinone electron acceptor of PSII (Q_A_). Six leaves were assessed per treatment. Replicate leaves were collected using individual plants.

### 4.8. Statistical Analysis

The experiments were arranged in a completely randomized design. Data were analyzed using SAS software (v. 9.4, SAS Institute Inc., Cary, NC, USA). Mean separations were calculated using Duncan’s multiple range tests at *p* ≤ 0.05.

## Figures and Tables

**Table 1:** Growth and morphology of chrysanthemum cuttings exposed for 30 days to different light quality regimes (red (R), red and blue (R:B), red, blue and far-red (R:B:FR), white (W) and blue (B); see the spectrum in Figure 9). During the experiment, the photosynthetic photon flux density was set to 150 ± 5 µmol m^−2^ s^−1^. Six replicates per treatment were assessed. Means within a column followed by the same letters are not significantly different at *p* ≤ 0.05, according to Duncan′s multiple range tests. CV % indicates the percentages of coefficient of variation among the treatments.

Spectrum	Cutting	Leaf			Shoot			Root		
	Survival (%)	Number (Plant^−1^)	Area (cm^2^ Leaf^−1^)	Area (cm^2^ Plant^−1^)	Fresh Weight (g)	Dry Weight (g)	Length (cm)	Fresh Weight (g)	Dry Weight (g)	Length (cm)
**R**	100	13.16 ^a^	3.48 ^c^	45.77 ^d^	7.04 ^c^	1.07 ^c^	15.12 ^ab^	0.54 ^b^	0.061 ^a^	4.04 ^a^
**R:B**	100	11.16 ^b^	8.58 ^a^	94.81 ^a^	9.80 ^b^	1.41 ^b^	13.61 ^bc^	0.77 ^a^	0.066 ^a^	5.01 ^a^
**R:B:FR**	100	11.17 ^b^	8.01 ^a^	88.71 ^ab^	9.40 ^b^	1.43 ^b^	16.31 ^a^	0.5 ^bc^	0.043 ^b^	4.38 ^a^
**W**	100	9.33 ^c^	7.65 ^ab^	70.67 ^c^	8.36 ^bc^	1.14 ^c^	13.41 ^bc^	0.43 ^bc^	0.03 ^b^	2.25 ^b^
**B**	100	12.16 ^ab^	6.93 ^b^	83.89 ^b^	11.79 ^a^	1.95 ^a^	12.12 ^c^	0.35 ^c^	0.04 ^b^	2.35 ^b^
**CV%**	0	10.62	12.17	7.8	17.1	14.91	11.83	27.12	27.91	27.25
***P***	-	0.0002	≤0.0001	≤0.0001	0.0004	≤0.0001	0.0022	0.0004	0.0003	≤0.0001

**Table 2:** Abbreviations and formulas of the parameters assessed in the current study.

**Basic parameters**		
**F_0_**	Minimum fluorescence when all PSII reaction centers (RCs) are open (O-step of OJIP transient)	F_50µs_
**F_J_**	Fluorescence intensity at the J-step (2 ms) of OJIP	F_2ms_
**F_I_**	Fluorescence intensity at the I-step (30 ms) of OJIP	F_30ms_
**Fluorescence parameters**		
**F_m_**	Maximum fluorescence, when all PSII RCs are closed (P-step of OJIP transient)	F_1s_ = F_p_
**F_v_**	Variable fluorescence of the dark-adapted leaf	F_m_ − F_0_
**V_J_**	Relative variable fluorescence at time 2 ms (J-step) after the start of an actinic light pulse	(F_J_ − F_0_)/(F_m_ − F_0_)
**V_I_**	Relative variable fluorescence at time 30 ms (I-step) after the start of an actinic light pulse	(F_30ms_ − F_0_)/(F_m_ − F_0_)
**F_v_/F_m_**	Maximal quantum yield of PSII photochemistry	1 − (F_0_/F_m_) = (F_m_ −F_0_)/F_m_ = φ_P0_ = TR_0_/ABS
**Quantum yields and efficiencies/probabilities**		
Ψ_E0_	The probability that a trapped exciton moves an electron into the electron transport chain beyond Q^−A^	ET_0_/TR_0_ = (1 − V_J_)
**φ_E0_**	The quantum yield of electron transport	[1 − (F_0_/F_m_)](1 − V_J_) = ET_0_/ABS
**φ_D0_**	Quantum yield of energy dissipation	F_0_/F_m_
**φ_PAV_**	Average (from time 0 to t_FM_) quantum yield for primary photochemistry	φ_P0_ (1 − V_J_) = φ_P0_ (S_M_/t_FM_)
**Specific energy fluxes (per Q_A_ reducing PSII RC)**		
**ABS/RC**	The specific energy fluxes per RC for energy absorption	M_0_ (1/V_J_)(1/φ_P0_)
**TR_0_/RC**	Trapped energy flux (leading to Q_A_ reduction) per RC	M_0_ (1/V_J_)
**ET_0_/RC**	Electron transport flux (further than Q_A_^−^) per RC	M_0_ (1/V_J_)(1 − V_J_)
**DI_0_/RC**	Dissipated energy flux	(ABS/RC) − (TR0 /RC)
**Performance indexes (products of terms expressing partial potentials at steps of energy bifurcations)**		
**PI_ABS_**	Performance index for the photochemical activity	[(γRC/1 − γRC)(φ_P0_/1 − φ_P0_)(ψ_E0_/1 − ψ_E0_)]

## Data Availability

The data presented in this study are available on request from the corresponding author.

## Supplementary Materials

The following supporting information can be downloaded at: https://www.mdpi.com/article/10.3390/plants11233337/s1. Figure S1. Shoot mass ratio of chrysanthemum cuttings exposed for 30 d to different light quality regimes (red (R), red and blue (R:B), red, blue and far-red (R:B:FR), white (W) and blue (B), Figure S2. Spider plot representation of relative values of the morphological, biochemical (A), and OJIP test parameters (B) from the fluorescence transient exhibited by leaves sampled from chrysanthemum cuttings exposed for 30 days to different light quality regimes (red (R), red and blue (R:B), red, blue and far-red (R:B:FR), white (W) and blue (B).

## Abbreviations

B	blue
CEA	controlled-environment agriculture
Chl	chlorophyll
CPPB	closed plant production systems
DW	dry weight
FW	fresh weight
OJIP	polyphasic chlorophyll fluorescence induction curve
PAR	photosynthetically active radiation
PPFD	photosynthetic photon flux density
PSII	photosystem II
R	red
R:B	red and blue
R:B:FR	red, blue and far red
RC	reaction center
RMR	root mass ratio
SMR	shoot mass ratio
W	white
