# Supplementary material for: Growth, Biomass Partitioning, and Photosynthetic Performance of Chrysanthemum Cuttings in Response to Different Light Spectra
Source: Plants (Basel). 2022 Dec 1;11(23):3337. doi: 10.3390/plants11233337 (PMC9735900; doi:10.3390/plants11233337)
Supplement: Supplementary file 1 [file plants-11-03337-s001.zip › plants-1941961-supplementary.pdf]

plants

MDPI

Supplementary figures

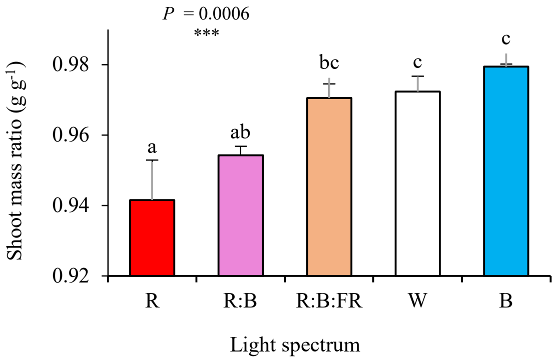

Figure S.1. Shoot mass ratio of Chrysanthemum cuttings exposed for 30 d to different light quality regimes (red (R), red and blue (R:B), red, blue and far-red (R:B:FR), white (W), and blue (B); see the spectrum in Figure 1). Photosynthetic photon flux density was set to 150 ± 5 µmol m^{-2} s^{-1} during the experiment. Six replicates per treatment were assessed. Bars represent SEM.

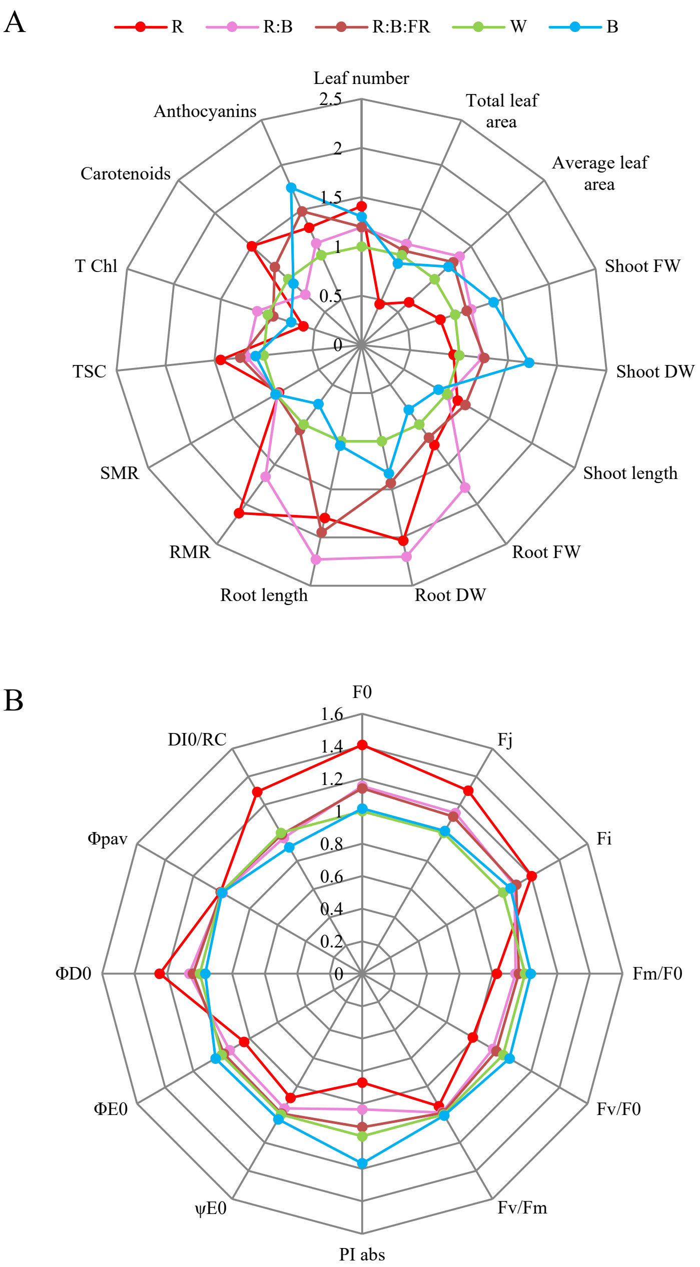

Figure S.2. Spider plot representation of relative values of the morphological, biochemical (A), and OJIP test parameters (B) from the fluorescence transient (equations and explanations in Table 2) exhibited by leaves sampled from Chrysanthemum cuttings exposed for 30 days to different light quality regimes (red (R), red and blue (R:B), red, blue and far-red (R:B:FR), white (W), as well as blue (B); see spectrum in Figure 1). Photosynthetic photon flux density was set to 150 ± 5 μmol m^{-2} s^{-1} during the experiment. Six replicate plants per treatment were assessed. Values are relative to W light. FW, fresh weight; DW, dry weight; RMR, root mass ratio (i.e., root DW/plant DW); SMR, shoot mass ratio (i.e., shoot DW/plant DW); TSC, total soluble carbohydrates; T Chl, total chlorophyll.
